# Psychological distress and unmet support needs among individuals at high hereditary cancer risk: Preliminary findings from a national needs assessment

**DOI:** 10.1192/j.eurpsy.2025.745

**Published:** 2025-08-26

**Authors:** E. Lowry, P. D’Alton, L. O’Driscoll, K. Cahill, D. McCartan, S. Deschênes

**Affiliations:** 1 University College Dublin; 2 St. Vincent’s University Hospital, Dublin, Ireland

## Abstract

**Introduction:**

Individuals at high familial or genetic risk of cancer face unique psychological challenges that can lead to significant distress, including worry, fear, and uncertainty about their future health and family members’ risk. This study assessed mental health challenges, including anxiety and depression, in this population and explored their preferred forms of psychological support.

**Objectives:**

To examine the prevalence of psychological distress in individuals at high hereditary cancer risk, the relationship between their current perceived support and distress, and preferred psychological support forms to inform the development of tailored mental health services.

**Methods:**

Participants (N = 152; 95% female, 3% male, 2% non-binary; mean age = 42.6 ± 8.3) were cancer-free residents of the Republic of Ireland identified as high-risk for hereditary cancer through high-risk clinic attendance or self-reported genetic screening or family history. They completed a cross-sectional survey using standardised mental health assessments, including Generalized Anxiety Disorder-7 (GAD-7) and Patient Health Questionnaire-9 (PHQ-9), and self-reported adequacy of psychological support and preferences. Descriptive statistics and post-hoc tests were used to analyse psychological distress, support adequacy, and preferences.

**Results:**

Moderate to severe anxiety (GAD-7 ≥10) was reported by 23%, and moderate to severe depression (PHQ-9 ≥10) by 18%. Participants reporting inadequate support had significantly higher anxiety (M = 8.1 ± 6.2) and depression (M = 6.4 ± 5.7) scores than those with adequate support (GAD-7: M = 4.2 ± 3.9; PHQ-9: M = 3.6 ± 3.9). ANOVA indicated significant differences in anxiety (F (2,132) = 7.04, p = .001) and depression (F (2,130) = 4.68, p = .011) scores across support levels. Post-hoc Tukey tests showed poorer mental health for those without support (p < .001 for anxiety; p = .013 for depression). Mental health professionals (psychologists and counsellors) were the top preferences for support, chosen by 74.8% of participants, with 47.5% selecting a psychologist and 27.3% a counsellor. An additional 12.2% preferred a healthcare professional (e.g., General Practitioner or nurse).

**Conclusions:**

This study provides important insights into the mental health challenges faced by individuals at high hereditary cancer risk, particularly those lacking adequate psychological support. Participants reporting inadequate support experienced significantly higher levels of anxiety and depression. These findings highlight the importance of ensuring adequate psychological support services for this population and their strong preference for professional psychological care. Integrating this care into routine genetic counselling and oncology services could help to address the unmet mental health needs of this underserved population.

**Disclosure of Interest:**

None Declared

